# The Apex Beat of the Heart

**Published:** 1908-04-18

**Authors:** 


### Diseases of Children.

### THE APEX BEAT OF THE HEART.

The apex beat is regarded as the lowest and most external point at which the cardiac impulse can be felt. Its position, if abnormal, is a valuable indication of cardiac or intra-thoracic mischief, but more so when the disease is on the left than on the right side. The reason of this difference in the relative value of alteration in the position of the apex is that normally in infants and young children the apex is situated more to the left and higher up than in later life. In infancy it is usually in the fourth space about half an inch outside the nipple line, and up to the fourth year an apex beat in the nipple line must be regarded as normal. Further, it seems that the heart is more movable inside the thorax than in later life. Thus, we frequently find that, when a child has been lying on the left side for some little time, the apex beat is considerably displaced outwards. In the examination of every patient a careful note should be made of the position of the apex at the beginning of an illness. Then, any subsequent alteration in position will be of considerable value in diagnosis.

Displacement of the apex may result from cardiac causes, from disease of the lungs or mediastinum, or from affections of the pleural cavity, such as effusions of all kinds, new growths, and diaphragmatic hernia. In addition, the heart is frequently displaced upwards and its apex considerably outwards as the result of gaseous distension of the stomach and intestines, ascites, or intra-abdominal tumours. An infrequent cause is transposition of the heart. It may occur independently or in association with transposition of other viscera, and may be compatible with prolonged life.

The common cardiac causes are hypertrophy and dilatation, often due to anæmia, pericardial effusion, rheumatic myocarditis, or overstrain after the heart-muscle has been weakened by illness of a toxic nature, such as influenza, diphtheria, and other specific fevers. An interesting case was that of a boy, aged four years, with signs of slight pleural effusion on the left side and an apex-beat in the fourth space on the left side an inch outside the nipple line. There was no cardiac murmur and nothing suggestive of rheumatism. While under observation the effusion was rapidly re-absorbed and the heart's apex receded to the nipple line in the fourth space. Three weeks later the boy had a definite mitral systolic murmur. Hence, this was almost certainly a case of rheumatic pleurisy with myocarditis. Had the effusion been on the right side it would undoubtedly have been assumed that the position of the apex was due to the presence of the fluid in the thorax.

In pleural effusions on the left side the position of the apex of the heart is a more valuable sign than when the effusion is on the right side. The displacement of the apex a little outwards is of slight diagnostic value, unless the displacement has taken place while the patient has been under observation and there is no evidence of cardiac dilatation. When, however, the heart is displaced to the right it is a much more obvious and striking physical sign which requires prompt explanation. Many patients are seen with a history of recent pneumonia which has not cleared up properly and has been, perhaps, followed by increasing shortness of breath. The position of the apex will at once afford a clue to the presence of pleural effusion. And, indeed, it is a most valuable sign in young children, for the physical signs over the lower half of the back of the chest may strongly suggest a pneumonic condition. It is quite common to find dulness, loud bronchial breathing, and bronchophony, although there is a considerable amount of effusion. This is partly due to compression of the lung, with retraction into the angular space at the side of the vertebral column, and sometimes partly due to an associated patchy broncho-pneumonia. Even if the fluid is purulent the physical signs may simulate those of consolidation. In large effusions there is little difficulty, for there are other signs such as expansion of the chest to the position of complete inspiration, deficient movement, dulness extending up to or above the clavicle, on to the sternum, and as far as the opposite edge of the manubrium, with feeble or absent air entry over the front of the chest. In small effusions the difficulty is much greater, for the displacement of the apex may be due to dilatation of the left ventricle. It is in cases of this kind that one gets assistance from carefully mapping out the position of the heart and from an examination with the $x$-rays, noting the position of the heart and the shape and movement of the diaphragm. Pneumothorax and diaphragmatic hernia are fortunately rare in the young, particularly in infancy. In pneumothorax there are all the signs of pleural effusion with hyper-resonance over the affected side. In diaphragmatic hernia the physical signs may be similar, but more often they vary in accordance with the nature of the intestinal contents, whether fluid or gaseous. At one time physical examination may yield dulness at the base, and at another the note may be hyper-resonant. This variability in the resonance is the most important diagnostic sign, taken in conjunction with displacement of the apex. In the absence of the dulness, the physical signs may be limited to a displaced apex, or the whole heart may be so pushed over to the right that the case is thought to be one of transposition. This is a natural error, as the percussion note may be resonant and the breath-sounds little altered, being readily transmitted from the compressed lung. More often the breath-sounds are feeble or absent. Then the conjunction of feeble breath-sounds and more or less normal resonance with a greatly displaced heart clinches the diagnosis. Solid tumours in the chest, whether mediastinal, pleural, or pulmonary, chiefly give rise to difficulty in diagnosis in the early stages, but usually it is not until the growth has reached a considerable size that it causes symptoms which attract attention.

